# Immune function of miR-214 and its application prospects as molecular marker

**DOI:** 10.7717/peerj.10924

**Published:** 2021-02-16

**Authors:** Qiuyuan Wang, Yang Liu, Yiru Wu, Jie Wen, Chaolai Man

**Affiliations:** College of Life Science and Technology, Harbin Normal University, Harbin, China

**Keywords:** miR-214, Immune cell, Tumor immunity, Inflammation, Virus, Molecular marker

## Abstract

MicroRNAs are a class of evolutionary conserved non-coding small RNAs that play key regulatory roles at the post-transcriptional level. In recent years, studies have shown that miR-214 plays an important role in regulating several biological processes such as cell proliferation and differentiation, tumorigenesis, inflammation and immunity, and it has become a hotspot in the miRNA field. In this review, the regulatory functions of miR-214 in the proliferation, differentiation and functional activities of immune-related cells, such as dendritic cells, T cells and NK cells, were briefly reviewed. Also, the mechanisms of miR-214 involved in tumor immunity, inflammatory regulation and antivirus were discussed. Finally, the value and application prospects of miR-214 as a molecular marker in inflammation and tumor related diseases were analyzed briefly. We hope it can provide reference for further study on the mechanism and application of miR-214.

## Introduction

MicroRNAs (miRNAs) are a kind of high conserved non-coding small RNAs in evolution that bind to the 3′-untranslated region (3′-UTR) of target gene mRNA and regulate gene expression at post-transcriptional level. In immune responses, miRNAs act as signal-regulating molecules after immune-related receptors activation, and affect the expression of immune-related genes, thus extensively participating in various aspects of immune response (Bosisio et al., 2019; Mehta & Baltimore, 2016).

**Figure 1:**
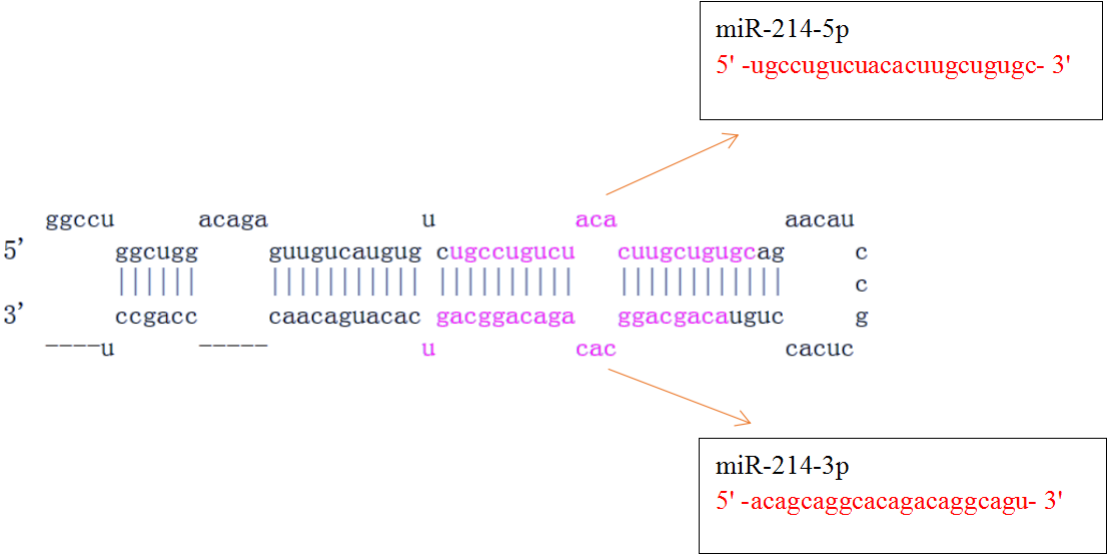
Pre-miR-214 and its mature miRNAs (miR-214-5p and miR-214-3p). (http://www.mirbase.org/cgi-bin/mirna_entry.pl?acc=MI0000290).

MiR-214, one of the key miRNAs involved in immune response, is widely distributed in fish, amphibians, birds, mammals and other vertebrates (Thomas, Adam & John, 2014). Hsa-miR-214 is located in the intron of *dynamin-3* gene and has-miR-199a is located about 6 kb next to miR-214, and miR-199a/miR-214 cluster often participates in regulating the same reactions. For example, miR-199a/miR-214 cluster can target *E-cadherin* and *claudin-2* and promote high glucose-induced peritoneal fibrosis (Lee et al., 2009; Che et al., 2017). In human, pre-miR-214 can encode two mature miRNAs: miR-214-5p and miR-214-3p, and miR-214-5p is hardly expressed, while miR-214-3p is high expressed based on 136 published RNA-seq experiments (http://www.mirbase.org/cgi-bin/mirna_entry.pl?acc=MI0000290), so there are functional differences between them (Bartel, 2004; Teng, Ji & Zhao, 2018; Li, Wang & Ren, 2018; Deng et al., 2019; Wang et al., 2019a; Wang et al., 2019b) (Fig. 1). Recently, a number of studies have reported the function and mechanism of miR-214 in the fields of immune response, tumor, cardiovascular, development, neurology, and metabolism, which has been a hotspot of miRNAs study. This review mainly focuses on the immune functions and application prospects of miR-214.

### Survey methodology

We used the PubMed database to search the keywords “miR-214”, combined with “immunity”, “inflammation”, “T cell”, “tumor immunity”, “virus”, “molecular marker” to obtain relevant articles and summarized them. Among them, miR-214 combined with “inflammation” retrieved 54 articles, combined with “immunity” retrieved 39 articles, combined with “T cell” retrieved eight articles, and “tumor immunity” retrieved 15 articles, combined with “virus” retrieved 33 articles. Finally, in these articles, we focused on screening the documents directly related to miR-214, and removed the non-key studies on miR-214 and documents not directly related to miR-214. The search was conducted in December 2018, a repeated search was conducted in October 2019, and a third repeated search was conducted in August 2020. The final reference time of our manuscript was from 1998 to 2020.

### MiR-214 and immune cells

MiR-214 regulates the functions and characteristics of a variety of immune cells including dendritic cells (DCs), T cells, natural killer (NK) cells, and macrophages, etc., and participates in immune response processes widely.

**Figure 2:**
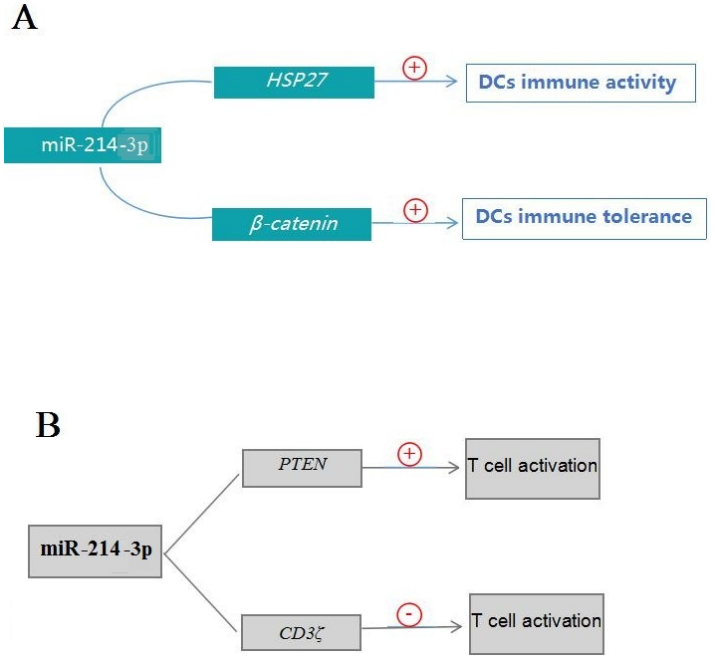
Pathways of miR-214-3p in regulating the functions of DCs and T cells. (A) The pathways of miR-214-3p in regulating DCs functions; (B) the pathways of miR-214-3p in regulating T cells activities; ⊕ indicates promotion, and ⊖ indicates inhibition.

MiR-214 is a key miRNA that regulates the functions of DCs. Studies have found that DCs immune activity is inhibited by regulatory T (Treg) cells, and the down-regulation of miR-214-3p can enhance the expression of heat shock protein 27 (HSP27) which inhibits the differentiation of Treg cells, so the overexpression of miR-214-3p can promote the differentiation of Treg cells and enhance its inhibitory effect on DCs immune activity (Pandey et al., 2013; Alexander & Steffen, 2014; Svajger & Rozman, 2014; Huan et al., 2017). Moreover, miR-214-3p can target the 3′UTR of the *β-catenin* which is a key regulator of DCs tolerance, down-regulation of miR-214-3p can induce DCs immune tolerance (Fändrich, 2010; Gordon et al., 2014; Gu et al., 2015). Therefore, miR-214-3p comprehensively affects the immune activity and tolerance of DCs by regulating the expression of *β-catenin* and differentiation of Treg cells (Fig. 2). In addition, tolerogenic DCs can promote central or peripheral tolerance through the deletion of T cells, induction of Tregs, expression of immunomodulatory molecules, and the production of immunosuppressive factors (Ilarregui et al., 2009; Ezzelarab & Thomson, 2011; Li & Shi, 2015), so miR-214 may have potential application value in the rejection of organ transplantation and the prevention and treatment of autoimmune diseases.

MiR-214 plays an important regulatory role in cellular immune response by regulating the activation, proliferation and differentiation of T cells. For example, PTEN protein, an inhibitor of the PI3K-AKT signaling pathway, negatively regulates T cell activation (Buckler, Liu & Turka, 2008). MiR-214-3p can target PTEN and improve the activity of PI3K-AKT signaling pathway, which promotes T cells activation (Jindra et al., 2010). In addition, the up-regulation of miR-214-3p in activated T cells can enhance the proliferation capacity of T cells (Jindra et al., 2010). Interestingly, miR-214-3p also inhibits T cell activation because T cell activation requires the signal integration and transduction by T cell receptor (TCR)-CD3 complex, and CD3*ζ* plays a key role in the signal transduction. MiR-214-3p can target the 3′-UTR of CD3*ζ* gene and negatively regulate T cell activation (Xiao et al., 2019). Therefore, miR-214-3p plays a key regulatory role in balancing the activation of T cells (Fig. 2). In addition, gga-miR-214 is significantly up-regulated in chicken thymus under immunosuppressive condition, but whether the up-regulation of gga-miR-214 is related to T cell suppression in thymus is worthy of further study (Zhou et al., 2019). It is worth mentioning that miR-214 also regulates the differentiation of certain T cells. MiR-214 plays a key role in the differentiation of naive T cells to Th17 cells during the relapse and remission phases of multiple sclerosis (MS) patients, because miR-214 may be a negative regulator of Th17 cell differentiation and the imbalance of Th17 cells is a key factor leading to MS (Brucklacher-Waldert et al., 2009). Therefore, miR-214 may have potential value in the prevention and treatment of MS (Ahmadian-Elmi et al., 2016).

MiR-214 plays an important role in regulating functions of NK cells and macrophages. NK cells are closely related to the occurrence and maintenance of pregnancy (Bezman et al., 2010; Nabila, 2019). MiR-214 is differentially expressed between uterus decidual and peripheral blood natural killer cells, which may affect the NK cells differentiation and the process of pregnancy (Carlino et al., 2018). So, in-depth research on miR-214 regulating NK cells functions can help to understand the pathological mechanisms of pregnancy injury at molecular level. It is well known that macrophages are important immune cells with phagocytic function and mediate the transition from innate immunity to adaptive immunity (Arango Duque & Descoteaux, 2014). Study shows that virus mimics polyriboinosinic polyribocytidylic acid (pIC) can induce up-regulation of 10 miRNAs expression in Atlantic cod macrophages, including miR-214-1-5p, suggesting miR-214 may play an important role in the antiviral immune response (Eslamloo et al., 2017).

In short, miR-214 plays an important regulatory role in immune activity and tolerance of DCs, proliferation and differentiation of T cells, and functions of NK cells and macrophages (Table 1). In-depth research on the functions and applications of miR-214 in immune cells may have positive theoretical and practical significances for the prevention and treatment of various diseases, such as organ transplantation, autoimmune diseases, pregnancy injury, immune tolerance and immunosuppression.

**Table 1:** Expressional changes and biological effects of miR-214 in immune and inflammatory responses.

**miR-214**	**Expressional change**	**Target gene**	**Biological effect**	**Reference**
miR-214-3p	Up	*HSP27*	Promote Tregs cell differentiation	Huan et al. (2017)
miR-214-3p	Down	*β-catenin*	Induce DCs tolerance and inhibit ovarian cancer	Gordon et al. (2014), Fändrich (2010) and Gu et al. (2015)
miR-214-3p	Up	*PTEN*	Promote T cell activation and proliferation, inhibit Tregs cell proliferation and tumor growth	Velasco et al. (2006), Jindra et al. (2010), Yin et al. (2014), Yang et al. (2008), Zhang et al. (2010) and Myers et al. (1998)
miR-214-3p	Up	*CD3ζ*	Inhibit T cell activation	Xiao et al. (2019)
miR-214-3p	Down	*PDL1*	Regulate T cells and further mediate the immune response of tumor cells	Song, Park & Uhm (2019), Sun, Zhang & Zhang (2019) and Wang et al. (2017)
miR-214-3p	Down	*B7-H3*	Regulate the polarization pathway of M2 macrophages	Fauci et al. (2012)
miR-214-3p	Up	*A2AR*	Amplify inflammatory effect	Zhao et al. (2015)
miR-214-5p	Up	*TSG-6*	Promote proinflammatory factor release	Hu et al. (2018)
miR-214-3p	Up	*TWIST1*	Promote aortic valve stromal cell calcification	Li et al. (2016a) and Li et al. (2016b)
miR-214-3p	Up	*MyD88*	Increase the secretion of pro-inflammatory factors and the number of calcified nodules	Zheng et al. (2019)
miR-214-3p	Down	*Mfn2*	Promote EMT process and bladder wall fibrosis, induce IC development	Lv et al. (2017)
miR-214-3p	Up	*TLR4*	Inhibit inflammation and cyst growth	Lakhia et al. (2020)

### MiR-214 and tumor immunity

MiR-214 plays a key regulatory role in the proliferation, invasion and metastasis of tumor cells (Yin et al., 2014). Recently, studies have found that Treg cells play an important role in mediating immune escape of tumor cells and have become important targets for tumor immunotherapy (Kasinski Andrea & Slack Frank, 2011; Ahmetlić et al., 2019). Interestingly, miR-214 expression is often up-regulated in several tumor cells (such as Gastric cancer Ji et al., 2019) and tumor-secreted miR-214 into recipient T cells by microvesicles (Yin et al., 2014). When miR-214-3p from microvesicles enters CD4^+^T cells and down-regulates PTEN, it can inhibit tumor through antagonizing phosphorylase activity, such as tyrosine kinase (Myers et al., 1998; Yang et al., 2008). In addition, the activities of Treg cells are often up-regulated in tumor patients, thereby promoting the development of tumors (Pandey et al., 2013; Alexander & Steffen, 2014; Svajger & Rozman, 2014). If miR-214-3p activity is blocked or down-regulated in microvesicles, then PTEN expression is up-regulated, Treg cells activity is down-regulated, and DCs immune activity is up-regulated, so the tumor growth may be inhibited. For example, Yin et al. (2014) used microvesicles to inoculate anti-miR-214 antisense oligonucleotides to tumor mice via tail vein, which significantly inhibited Treg cells proliferation and tumor growth, indicating that the potential application of miR-214 for tumor micro-regulation of Treg cells (Yin et al., 2014) (Table 1).

Programmed cell death receptor ligand 1 (PD-L1) interacting with programmed cell death protein 1 (PD-1) can inhibit T cell activation, induce effector T cell apoptosis, finally inhibit tumor immunity , which is important for using immunotherapy to treat tumors (Dong et al., 1999; Ceeraz, Nowak & Noelle, 2013; Zhang, Medeiros & Young, 2018; Wang et al., 2019a; Wang et al., 2019b). MiR-214-3p can inhibit the expression of PD-L1 by targeting its 3′UTR, but lncRNA urothelial carcinoma associated 1 (UCA1) can up-regulate PD-L1 expression through inhibiting miR-214-3p, which promotes gastric cancer (GC) cell proliferation and migration and inhibits apoptosis, so miR-214-3p can be used as potential new therapeutic target of GC treatment (Wang et al., 2017; Sun, Zhang & Zhang, 2019; Song, Park & Uhm, 2019). In diffuse large b-cell lymphoma (DLBCL), miR-214 also targets PD-L1 to regulate T cells, and mediates the immune response of tumor cells (Song, Park & Uhm, 2019) (Table 1).

Multiple myeloma (MM) is related to macrophage polarization. In MM patients, lncRNA nuclear paraspeckle assembly transcript 1 (NEAT1) and B7-H3 are up-regulated, but miR-214 is significantly down-regulated (Fauci et al., 2012; Wang, Kang & Shan, 2014; Mao et al., 2017). NEAT1 can directly target miR-214, and miR-214 directly binds to B7-H3. If NEAT1 is silenced, miR-214 will inhibit the expression of B7-H3, thus inhibiting the polarization of M2 macrophages by inhibiting JAK2/STAT3 signaling (Gao et al., 2020). Therefore, miR-214 plays an important role in the polarization pathway of MM related M2 macrophages (Table 1).

With the deep-going of the research, we believe tumor therapeutic agents including miR-214 will be developed in the future. Therefore, the thorough understanding of the molecular mechanisms of miR-214, the development of new preparations, the molecular mechanism of tumor treatment, and the feasibility of treatment may be issues that need to solve urgently at this stage.

### MiR-214 and inflammation

MiR-214 plays a key regulatory role in promoting inflammation. For example, adenosine A2A receptors (A2ARs) have anti-inflammatory effects, and up-regulation of miR-214 expression in inflammatory cells can inhibit adenosine A2AR expression. Moreover, the decrease of A2AR expression weakens the inhibition of nuclear factor kappa-B (NF-*κ*B) through PKA, which promotes the up-regulation of miR-214-3p to amplify the inflammatory response (Zhao et al., 2015). In addition, miR-214 also promotes the release of inflammatory factors. For example, Adipose-derived stem cells (ADSCs) inhibit the inflammation of microglia by secreting tumor necrosis factor-inducible gene 6 protein (TSG-6). TSG-6 inhibits the release of pro-inflammatory factors such as IL-1*β*, IL-6 and TNF*α*, while *TSG-6* is negatively regulated by miR-214-5p, so miR-214-5p can increase the release of proinflammatory factors by inhibiting TSG-6 (Hu et al., 2018). Furthermore, miR-214 plays a key regulatory role in inflammatory response and calcification of human aortic valve interstitial cells (AVICs). M1 macrophages transmit miR-214 to valvular interstitial cells through microvesicles, miR-214-3p directly targets Twist1 and down-regulates it, thus promoting calcification of valve interstitial cells (Li et al., 2016a; Li et al., 2016b). Further research finds that miR-214 is related to the expression level of MyD88 protein. Up-regulation of miR-214-3p promotes the expression of MyD88 and NF-*κ*B, while the up-regulation of MyD88 increases the secretion of pro-inflammatory factors and the number of calcified nodules (Zheng et al., 2019). Another, miR-214-3p can be selectively inhibited by 17*β*-estradiol (E2) or progesterone (P), while E2 and P can indirectly inhibit apoptosis and inflammation-related gene translation. So it is speculated that E2 and P suppress inflammation by inhibiting miR-214-3p expression (Herzog et al., 2016). Besides, miR-214-3p is down- expressed in the post-menopausal women’s epithelial-mesenchymal transition (EMT) process and the development of interstitial cystitis (IC), and mitofusin 2 (Mfn2) is the target gene of miR-214-3p, so down-regulation of miR-214-3p promotes the EMT process and bladder wall fibrosis, leading to IC in postmenopausal women (Lv et al., 2017). However, miR-214-3p is up- regulated in the kidney, pancreas and serum of hyperlipidemic pancreatitis (HP) rats. Up- regulation of miR-214-3p inhibits PTEN expression, but increases the level of P-Akt in HP kidneys which may be a possible mechanism for inducing severe symptoms of pancreatitis, and exacerbates HP-induced pathological changes, kidney and pancreas damage, and fibrosis. Therefore, using miR-214-3p as target for the treatment of acute renal injury of HP provides a potential and effective method for the clinic (Yan et al., 2020).

Interestingly, miR-214 also plays a regulatory role in inhibiting inflammation. Substantial interstitial inflammation caused by renal cysts is often ignored, and miR-214 is up-regulated in cystic kidney of autosomal dominant polycystic kidney disease (ADPKD) patients (Lakhia et al., 2020). The up-regulation mechanism of miR-214 is mainly because of the pro-inflammatory TLR4/IFN-*γ*/STAT1 pathways activating the miR-214 host gene (Watanabe et al., 2008). In turn, miR-214-3p targets TLR4, and inhibits the inflammatory response. Therefore, the up-regulation of miR-214-3p is a compensatory protective response of the cyst microenvironment, which can inhibit inflammation and cyst growth.

In summary, miR-214 plays different roles in multiple inflammatory response pathways (Table 1). Studying the pro-inflammatory and anti-inflammatory functions of miR-214 can provide positive theoretical basis for understanding the molecular mechanism of inflammatory response and developing new strategies for the diagnosis and treatment of inflammatory diseases.

### MiR-214 and virus

MiRNAs directly target RNA virus genes or affect the replication and pathogenesis of virus through altering the host transcriptome (Trobaugh & Klimstra, 2017). It is found that miR-214 is differentially expressed in virus-infected tissues. For example, the expression of miR-214-3p is up-regulated in the plasma and myocardial cells of patients with viral myocarditis (VM) infected with coxsackievirus, but the specific mechanism is still unclear (Chen et al., 2015). Coxsackie adenovirus receptor (CAR) protein is an adenovirus receptor, and miR-214-3p can inhibit adenovirus replication by targeting the 3′-untranslated region of early region 1A (E1A) mRNA (Yanagawa-Matsuda et al., 2012). Therefore, in-depth study of the mechanism and biological effects of miR-214 in virus-infected tissues may have important theoretical and practical significance for the prevention and treatment of viral diseases including viral myocarditis.

Recent studies have found that miR-214 can inhibit the replication of fish viruses. For instance, miR-214-3p effectively inhibits siniperca chuatsi rhabdovirus (SCRV) replication, which may provide a new approach for the development of effective SCRV infection prevention strategies (Zhao et al., 2019). In addition, miR-214-3p also targets the coding regions of viral N and P to inhibit snakehead vesiculovirus (SHVV) replication (Zhang et al., 2017). Another study discovers that miR-214-3p can also target glycogen synthase (GS) gene and inhibit SHVV replication, because GS gene is the key gene for SHVV replication. So, miR-214-3p can inhibit SHVV replication from multiple aspects through multiple target genes, which provides several possibilities for the prevention and control of SHVV (Zhang et al., 2019).

### MiR-214 and molecular markers

In recent years, the application value of miR-214 as a diagnostic marker has attracted widespread attention.

In inflammatory diseases, miR-214 is one of the three lowest expressed miRNAs in gingival tissue inflammation in Japanese dental patients. It can be determined that abnormal expression of miR-214 is associated with chronic periodontitis, which provides a basis for the diagnosis of periodontal inflammatory diseases (Ogata et al., 2014). MiR-214 also is used as a non-invasive biomarker for the diagnosis of ankylosing spondylitis (AS). The expression level of miR-214 in serum of AS patients is significantly lower than that of normal people, and which is significantly correlated with the active C-reactive protein (CRP) of AS disease. Therefore, miR-214 as a diagnostic marker of AS disease provides a powerful help for the treatment and prevention of AS (Kook et al., 2019). Up-regulation of STAT6 promotes the secretion of proinflammatory cytokines in intestinal epithelial cells, and then participates in the inflammation response to induce ulcerative colitis (UC) (Rosen et al., 2011). *STAT6* is a direct target of miR-214-3p, so targeting *STAT6* pathway by miR-214-3p may become a new therapeutic target for UC (Li et al., 2017).

In tumor diseases, the expression of miR-214-3p in the plasma of gastric cancer (GC) patients is significantly higher than that of normal people, and GC patients with high miR-214 expression may have larger tumor lymphatic metastasis and tumor node metastasis (TNM) stage, higher levels of CEA (Carcinoembryonic antigen) and carbohydrate antigen 19-9 (CA19-9), and the survival rate is low. The high sensitivity and specificity of miR-214-3p for GC have high application value in the diagnosis and prognosis of GC (Zhang et al., 2015; Ji et al., 2019). In addition, miR-214 expression is down-regulated in human cholangiocarcinoma exosomes, sinonasal inverted papilloma (SNIP), difuse large B cell lymphoma (DLBCL) and bladder cancer (BC), which suggests that miR-214 has potential value as a molecular marker and therapeutic target (Xie et al., 2017; Kitdumrongthum et al., 2018; Teng et al. 2018; Sun, Zhang & Zhang, 2019). In breast cancer, the proliferation and migration ability of tumor cells with over-expressed miR-214-3p declines, and the cells are induced to apoptosis and interfered with the cell cycle (Liu et al., 2016). Similarly, the expression of miR-214-5p also decreases in hepatocellular carcinoma (HCC) tissues and cells. The over-expression of miR-214-5p can decrease cell proliferation, reduce cell migration, and block the cell cycle in G0/G1 phase (Pang et al., 2018). Above results indicate that miR-214 plays a key role in inhibiting breast cancer and HCC, and may become a potential biomarker and therapeutic target. MiR-214 is significantly down-regulated in esophageal squamous cell carcinoma (ESCC), and over-expression of miR-214 may impair the invasion and migration ability of Eca109, TE1 and KYSE150 cells. Therefore, miR-214 may have potential application value as a diagnostic marker and therapeutic target of ESCC (Lu et al., 2016). In addition, miR-214 expression is down-regulated in colon cancer tissues and cells. MiR-214-3p can inhibit the cell viability and development of colon cancer by inhibiting ADP-ribosylation factor-like protein 2 (ARL2) and mitochondrial transcription factor A (TFAM) (Long et al., 2015; Wu et al., 2018a; Wu et al., 2018b). So, miR-214 may be an important target for the treatment of colon cancer.

In other diseases, miR-214-3p expression is up-regulated in chronic kidney disease. Mitochondrial dysfunction is related to the pathogenesis of chronic kidney disease. MiR-214-3p has a pathogenic role in chronic kidney disease by disrupting mitochondrial oxidative phosphorylation, so miR-214-3p has the potential to become a therapeutic target and diagnostic biomarker for chronic kidney diseases such as nephritis (Bai et al., 2019). In addition, 6 miRNAs including miR-214-3p are found to be dysregulated in diabetic kidney disease (DKD), and these miRNAs are involved in the pathogenesis of apoptosis, fibrosis, and accumulation of extracellular matrix related to the pathogenesis of DKD, which indicates that miR-214-3p may have the potential to represent the disease biomarker (Assmann et al., 2018). In addition, miR-214-3p is significantly up-regulated in the pathogenesis of myocardial ischemia/reperfusion (I/R) injury, which provides new targets for myocardial I/R damage (Wang et al., 2016). MiR-214 also plays an important role in fibrotic diseases. Increasing the expression of miR-214-3p reduces the expression of collagen *α*l and connective tissue growth factor (CTGF) in endometriosis matrix and endometrial epithelial cells, which provides another treatment for endometrium heterotopic fibrosis (Wu et al., 2018a; Wu et al., 2018b). Interestingly, miR-214 also plays an important role in musculoskeletal metabolism, bone formation, and other bone diseases. Specifically, miR-214-3p mediates skeletal muscle myogenesis and the proliferation, migration and differentiation of vascular smooth muscle cells. MiR-214-3p also regulates bone formation by targeting specific molecular pathways and expression of various osteoblast-related genes (Sun et al., 2018). For example, osteoclast-derived exosome miR-214-3p transferred to osteoblasts can inhibit bone formation (Li et al., 2016a; Li et al., 2016b). MiR-214’s role in primary osteoporosis may be through inhibiting the expression of osterix to inhibit bone formation (Mohamad et al., 2019). So miR-214 may be an important potential target for the treatment of bone diseases.

In brief, more and more studies have shown the potential values and application prospects of miR-214 as a diagnostic marker in diseases such as inflammation and tumor (Table 2). It is believed that in the near future, miR-214 will truly appear in clinical practice detection as a diagnostic marker and play its due value for the diagnosis and treatment of clinically relevant diseases.

**Table 2:** Biological functions of miR-214 as a molecular marker.

**miRNA**	**Related disease**	**Expressional change**	**Application prospect**	**Reference**
miR-214-3p	Periodontitis	Down	Biomarker for the diagnosis of periodontitis-related diseases	Ogata et al. (2014)
miR-214	Ankylosing spondylitis	Down	Non-invasive biomarker for the diagnosis of ankylosing spondylitis	Kook et al. (2019)
miR-214-3p	Ulcerative colitis	Down	Therapeutic target for ulcerative colitis	Rosen et al. (2011)
miR-214-3p	Gastric cancer	Up	Biomarker value in the diagnosis and prognosis of gastric cancer	Zhang et al. (2015) and Ji et al. (2019)
miR-214-3p	Cholangiocarcinoma	Down	Biomarker for the diagnosis and treatment of cholangiocarcinoma	Kitdumrongthum et al. (2018)
miR-214-3p	Bladder cancer	Down	A potential therapeutic target for the treatment of bladder cancer	Xie et al. (2017)
miR-214-3p	Difuse large B cell lymphoma	Down	Biomarker for good prognosis of difuse large B cell lymphoma	Sun, Zhang & Zhang (2019)
miR-214-3p	Sinonasal inverted papilloma	Down	Biomarker for the diagnosis and treatment of sinonasal inverted papilloma	Teng et al. (2018)
miR-214-5p	Hepatocellular carcinoma	Down	Potential biomarker and therapeutic target for HCC	Pang et al. (2018)
miR-214-3p	Esophageal squamous cell carcinoma	Down	Potential diagnostic marker and therapeutic target of ESCC	Lu et al. (2016)
miR-214-3p	Colon cancer	Down	Potential target for the treatment of colon cancer	Long et al. (2015), Wu et al. (2018a) and Wu et al. (2018b)
miR-214-3p	Nephritis	Up	Therapeutic target and diagnostic biomarker for chronic kidney disease	Bai et al. (2019)
miR-214-3p	Diabetic kidney disease	Up	Potential diagnostic marker for diabetic kidney disease	Assmann et al. (2018)
miR-214-3p	Ischemia/reperfusion	Up	Biomarker for the diagnosis and treatment of myocardial I/R injury prevention	Wang et al. (2016)
miR-214-3p	Fibrotic diseases	Up	Potential target for treatment of endometrium heterotopic fibrosis	Wu et al. (2018a) and Wu et al. (2018b)
miR-214-3p	bone diseases.	Up	Potential target for the treatment of bone diseases	Sun et al. (2018) and Mohamad et al. (2019)

## Conclusions

With the deepening of research, the function and mechanism of miR-214 in the fields of immune cell regulation, inflammatory response, tumor immunity and virus replication are gradually revealed. Moreover, the potential clinical application value of miR-214 as a biomarker has attracted increasing attention. According to the research status, miR-214 has promising prospects in the following aspects: Firstly, miR-214 may have in-depth research value in the prevention and treatment of diseases such as organ transplant rejection, autoimmune disease and immune tolerance; Secondly, miR-214 regulates tumor microenvironment to make it have the ability to inhibit the immune escape of tumor cells and its potential application value; Thirdly, abnormal expression of miR-214 can affect the replication of several viruses, which indicates that miR-214 has good development prospect in the diagnosis and treatment of certain viral diseases; Finally, miR-214 has potential application value as a diagnostic marker and therapeutic target in multiple diseases. In short, deep study on the regulatory relationship and molecular regulatory mechanism of miR-214 not only provides important theoretical basis for scientific issues such as immune regulation, tumor treatment, inflammation diagnosis, and antivirus, but also paves the way for actively developing new strategies for the prevention and treatment of these diseases. It is believed that miR-214 will have great research value and bright application prospects whether it is used as a drug target for disease treatment or as a molecular marker for disease diagnosis and prognosis.

## Additional Information and Declarations

### Data Availability

The following information was supplied regarding data availability: This is a literature review; there is no data.

## References

[ref-1] Ahmadian-Elmi M, Bidmeshki Pour A, Naghavian R, Ghaedi K, Tanhaei S, Izadi T, Nasr-Esfahani MH (2016). miR-27a and miR-214 exert opposite regulatory roles in Th17 differentiation via mediating different signaling pathways in peripheral blood CD4(+) T lymphocytes of patients with relapsing-remitting multiple sclerosis. Immunogenetics.

[ref-2] Ahmetlić F, Riedel T, Hömberg N, Bauer V, Trautwein N, Geishauser A, Sparwasser T, Stevanović S, Röcken M, Mocikat R (2019). Regulatory T cells in an endogenous mouse lymphoma recognize specific antigen peptides and contribute to immune escape. Cancer Immunology Research.

[ref-3] Alexander M, Steffen J (2014). Development and function of dendritic cell subsets. Immunity.

[ref-4] Arango Duque G, Descoteaux A (2014). Macrophage cytokines: involvement in immunity and infectious diseases. Frontiers in Immunology.

[ref-5] Assmann TS, Recamonde-Mendoza M, De Souza BM, Bauer AC, Crispim D (2018). MicroRNAs and diabetic kidney disease: systematic review and bioinformatic analysis. Molecular and Cellular Endocrinology.

[ref-6] Bai M, Chen H, Ding D, Song R, Lin J, Zhang Y, Guo Y, Chen S, Ding G, Zhang Y, Jia Z, Huang S, He JC, Yang L, Zhang A (2019). MicroRNA-214 promotes chronic kidney disease by disrupting mitochondrial oxidative phosphorylation. Kidney International.

[ref-7] Bartel DP (2004). MicroRNAs: genomics, biogenesis, mechanism, and function. Cell.

[ref-8] Bezman NA, Cedars E, Steiner DF, Blelloch R, Hesslein DG, Lanier LL (2010). Distinct requirements of microRNAs in NK cell activation, survival, and function. Journal of Immunology.

[ref-9] Bosisio D, Gianello V, Salvi V, Sozzani S (2019). Exteracellular miRNAs as activators of innate immune receptors. Cancer Letters.

[ref-10] Brucklacher-Waldert V, Stuerner K, Kolster M, Wolthausen J, Tolosa E (2009). Phenotypical and functional characterization of T helper 17 cells in multiple sclerosis. Brain.

[ref-11] Buckler JL, Liu X, Turka LA (2008). Regulation of T-cell responses by PTEN. Immunological Reviews.

[ref-12] Carlino C, Rippo MR, Lazzarini R, Monsurrò V, Morrone S, Angelini S, Trotta E, Stabile H, Bastianelli C, Albertini MC, Olivieri F, Procopio A, Santoni A, Gismondi A (2018). Differential microRNA expression between decidual and peripheral blood natural killer cells in early pregnancy. Human Reproduction.

[ref-13] Ceeraz S, Nowak EC, Noelle RJ (2013). B7 family checkpoint regulators in immune regulation and disease. Trends in Immunology.

[ref-14] Chen ZG, Liu H, Zhang JB, Zhang SL, Zhao LH, Liang WQ (2015). Upregulated microRNA-214 enhances cardiac injury by targeting ITCH during coxsackievirus infection. Mol Med Rep.

[ref-15] Che M, Shi T, Feng S, Li H, Zhang X, Feng N, Lou W, Dou J, Tang G, Huang C, Xu G, Qian Q, Sun S, He L, Wang H (2017). The microRNA-199a/214 cluster targets e-cadherin and claudin-2 and promotes high glucose-induced peritoneal fibrosis. Journal of the American Society of Nephrology.

[ref-16] Deng ZF, Zheng HL, Chen JG, Luo Y, Xu JF, Zhao G, Lu JJ, Li HH, Gao SQ, Zhu LQ, Zhang YH, Wang F (2019). MiR-214-3p targets *β*-catenin to regulate depressive-like behaviors induced by chronic social defeat stress in mice. Cerebral cortex.

[ref-17] Dong H, Zhu G, Tamada K, Chen L (1999). B7-H1, a third member of the B7 family, co-stimulates T-cell proliferation and interleukin-10 secretion. Nature Medicine.

[ref-18] Eslamloo K, Inkpen SM, Rise ML, Andreassen R (2017). Discovery of microRNAs associated with the antiviral immune response of Atlantic cod macrophages. Molecular Immunology.

[ref-19] Ezzelarab M, Thomson AW (2011). Tolerogenic dendritic cells and their role in transplantation. Seminars in Immunology.

[ref-20] Fändrich F (2010). Cell therapy approaches aiming at minimization of immunosuppression in solid organ transplantation. Current Opinion in Organ Transplantation.

[ref-21] Fauci JM, Straughn Jr JM, Ferrone S, Buchsbaum DJ (2012). A review of B7-H3 and B7-H4 immune molecules and their role in ovarian cancer. Gynecologic Oncology.

[ref-22] Gao Y, Fang P, Li WJ, Zhang J, Wang GP, Jiang DF, Chen FP (2020). LncRNA NEAT1 sponges miR-214 to regulate M2 macrophage polarization by regulation of B7-H3 in multiple myeloma. Molecular Immunology.

[ref-23] Gordon JR, Ma Y, Churchman L, Gordon SA, Dawicki W (2014). Regulatory dendritic cells for immunotherapy in immunologic diseases. Frontiers in Immunology.

[ref-24] Gu C, Zhou XD, Yuan Y, Miao XH, Liu Y, Ru YW, Li KQ, Li G (2015). MicroRNA-214 induces dendritic cell switching from tolerance to immunity by targeting *β*-Catenin signaling. International Journal of Clinical and Experimental Pathology.

[ref-25] Herzog R, Zendedel A, Lammerding L, Beyer C, Slowik A (2016). Impact of 17beta-estradiol and progesterone on inflammatory and apoptotic microRNA expression after ischemia in a ratmodel. Journal of Steroid Biochemistry and Molecular Biology.

[ref-26] Hu Y, Li G, Zhang Y, Liu N, Zhang P, Pan C, Nie H, Li Q, Tang Z (2018). Upregulated TSG-6 expression in ADSCs inhibits the BV2 microglia-mediated inflammatory response. BioMed Research International.

[ref-27] Huan Y, He Y, Liu B, Li Y, Jia L, Qu C, Lv B, Zhang X, Peng H (2017). Zhenbao Pill reduces the percentage of Treg cells by inducing HSP27 expression. Biomedicine and Pharmacotherapy.

[ref-28] Ilarregui JM, Croci DO, Bianco GA, Toscano MA, Salatino M, Vermeulen ME, Geffner JR, Rabinovich GA (2009). Tolerogenic signals delivered by dendritic cells to T cells through a galectin-1-driven immunoregulatory circuit involving interleukin 27 and interleukin 10. Nature Immunology.

[ref-29] Ji B, Huang Y, Gu T, Zhang L, Li G, Zhang C (2019). Potential diagnostic and prognostic value of plasma long noncoding RNA LINC00086 and miR-214 expression in gastric cancer. Cancer Biomark.

[ref-30] Jindra PT, Bagley J, Godwin JG, Iacomini J (2010). Costimulation-dependent expression of microRNA-214 increases the ability of T cells to proliferate by targeting PTEN. Journal of Immunology.

[ref-31] Kasinski Andrea L, Slack Frank J (2011). Epigenetics and genetics, MicroRNAs enroute to the clinic: progress in validating and targeting microRNAs for cancer therapy. Nature Reviews Cancer.

[ref-32] Kitdumrongthum S, Metheetrairut C, Charoensawan V, Ounjai P, Janpipatkul K, Panvongsa W, Weerachayaphorn J, Piyachaturawat P, Chairoungdua A (2018). Dysregulated microRNA expression profiles in cholangiocarcinoma cell-derived exosomes. Life Sciences.

[ref-33] Kook HY, Jin SH, Park PR, Lee SJ, Shin HJ, Kim TJ (2019). Serum miR-214 as a novel biomarker for ankylosing spondylitis. International Journal of Rheumatic Diseases.

[ref-34] Lakhia R, Yheskel M, Flaten A, Ramalingam H, Aboudehen K, Ferrè S, Biggers L, Mishra A, Chaney C, Wallace DP, Carroll T, Igarashi P, Patel V (2020). Interstitial microRNA miR-214 attenuates inflammation and polycystic kidney disease progression. JCI Insight.

[ref-35] Lee YB, Bantounas I, Lee DY, Phylactou L, Caldwell MA, Uney JB (2009). Twist-1 regulates the miR-199a/214 cluster during development. Nucleic Acids Research.

[ref-36] Li D, Liu J, Guo B, Liang C, Dang L, Lu C, He X, Cheung HY, Xu L, Lu C, He B, Liu B, Shaikh AB, Li F, Wang L, Yang Z, Au DW, Peng S, Zhang Z, Zhang BT, Pan X, Qian A, Shang P, Xiao L, Jiang B, Wong CK, Xu J, Bian Z, Liang Z, Guo DA, Zhu H, Tan W, Lu A, Zhang G (2016a). Osteoclast-derived exosomal miR-214-3p inhibits osteoblastic bone formation. Nature Communications.

[ref-37] Li H, Shi B (2015). Tolerogenic dendritic cells and their applications in transplantation. Cellular & Molecular Immunology.

[ref-38] Li HD, Wang HQ, Ren Z (2018). MicroRNA-214-5p inhibits the invasion and migration of hepatocellular carcinoma cells by targeting wiskott-aldrich syndrome like. Cellular Physiology and Biochemistry.

[ref-39] Li JA, Wang YD, Wang K, Wang ZL, Jia DY, Yang BY, Xiong CB (2017). Downregulation of miR-214-3p may contribute to pathogenesis of ulcerative colitis via targeting STAT6. BioMed Research International.

[ref-40] Li XF, Wang Y, Zheng DD, Xu HX, Wang T, Pan M, Shi JH, Zhu JH (2016b). M1 macrophages promote aortic valve calcification mediated by microRNA-214/TWIST1 pathway in valvular interstitial cells. American Journal of Translational Research.

[ref-41] Liu B, Tian Y, Li F, Zhao Z, Jiang X, Zhai C, Han X, Zhang L (2016). Tumor-suppressing roles of miR-214 and miR-218 in breast cancer. Oncology Reports.

[ref-42] Long LM, He BF, Huang GQ, Guo YH, Liu YS, Huo JR (2015). microRNA-214 functions as a tumor suppressor in human colon cancer via the suppression of ADP-ribosylation factor-like protein 2. Oncology Letters.

[ref-43] Lu Q, Xu L, Li C, Yuan Y, Huang S, Chen H (2016). MiR-214 inhibits invasion and migration via downregulating GALNT7 in esophageal squamous cell cancer. Tumour Biology.

[ref-44] Lv JW, Wen W, Jiang C, Fu QB, Gu YJ, Lv TT, Li ZD, Xue W (2017). Inhibition of microRNA-214 promotes epithelial-mesenchymal transition process and induces interstitial cystitis in postmenopausal women by upregulating Mfn2. Experimental & Molecular Medicine.

[ref-45] Mao Y, Chen L, Wang F, Zhu D, Ge X, Hua D, Sun J (2017). Cancer cell-expressed B7-H3 regulates the differentiation of tumor-associated macrophages in human colorectal carcinoma. Oncology Letters.

[ref-46] Mehta A, Baltimore D (2016). MicroRNAs as regulatory elements in immune system logic. Nature Reviews Immunology.

[ref-47] Mohamad N, Nabih ES, Zakaria ZM, Nagaty MM, Metwaly RG (2019). Insight into the possible role of miR-214 in primary osteoporosis via osterix. Journal of Cellular Biochemistry.

[ref-48] Myers MP, Pass I, Batty IH, Van der Kaay J, Stolarov JP, Hemmings BA, Wigler MH, Downes CP, Tonks NK (1998). The lipid phosphatase activity of PTEN is critical for its tumor supressor function. Proceedings of the National Academy of Sciences of the United States of America.

[ref-49] Nabila JF (2019). Features of human decidual NK cells in healthy pregnancy and during viral infection. Frontiers in Immunology.

[ref-50] Ogata Y, Matsui S, Kato A, Zhou L, Nakayama Y, Takai H (2014). MicroRNA expression in inflamed and noninflamed gingival tissues from Japanese patients. Journal of Oral Science.

[ref-51] Pandey G, Cohain A, Miller J, Merad M (2013). Decoding dendritic cell function through module and network analysis. Journal of Immunological Methods.

[ref-52] Pang J, Li Z, Wang G, Li N, Gao Y, Wang S (2018). miR-214-5p targets KLF5 and suppresses proliferation of human hepatocellular carcinoma cells. Journal of Cellular Biochemistry.

[ref-53] Rosen MJ, Frey MR, Washington MK, Chaturvedi R, Kuhnhein LA, Matta P, Revetta FL, Wilson KT, Polk DB (2011). STAT6 activation in ulcerative colitis: a new target for prevention of IL-13-induced colon epithelial cell dysfunction. Inflammatory Bowel Disease.

[ref-54] Song MK, Park BB, Uhm J (2019). Understanding immune evasion and therapeutic targeting associated with PD-1/PD-L1 pathway in diffuse large B-cell lymphoma. International Journal of Molecular Sciences.

[ref-55] Sun Y, Kuek V, Liu Y, Tickner J, Yuan Y, Chen L, Zeng Z, Shao M, He W, Xu J (2018). MiR-214 is an important regulator of the musculoskeletal metabolism and disease. Journal of Cellular Physiology.

[ref-56] Sun JR, Zhang X, Zhang Y (2019). MiR-214 prevents the progression of diffuse large B-cell lymphoma by targeting PD-L1. Cellular & Molecular Biology Letters.

[ref-57] Svajger U, Rozman P (2014). Tolerogenic dendritic cells: molecular and cellular mechanisms in transplantation. Journal of Leukocyte Biology.

[ref-58] Teng JW, Ji PF, Zhao ZG (2018). MiR-214-3p inhibits *β*-catenin signaling pathway leading to delayed fracture healing. European Review for Medical and Pharmacological Sciences.

[ref-59] Teng Y, Li Y, Lin Z, Gao Y, Cao X, Lou X, Lin F, Li Y (2018). Analysis of miRNA expression profiling identifies miR-214-3p as a novel biomarker in sinonasal inverted papilloma. Epigenomics.

[ref-60] Thomas D, Adam C, John HP (2014). Evolution of the miR199-214 cluster and vertebrate skeletal development. RNA Biology.

[ref-61] Trobaugh DW, Klimstra WB (2017). MicroRNA regulation of RNA virus replication and pathogenesis. Trends in Molecular Medicine.

[ref-62] Velasco A, Bussaglia E, Pallares J, Dolcet X, Llobet D, Encinas M, Llecha N, Palacios J, Prat J, Matias-Guiu X (2006). PIK3CA gene mutations in endometrial carcinoma. Correlation with PTEN and K-RAS alterations. Human Pathology.

[ref-63] Wang H, Guan Z, He K, Qian J, Cao J, Teng L (2017). LncRNA UCA1 in anti-cancer drug resistance. Oncotarge.

[ref-64] Wang X, Ha T, Hu Y, Lu C, Liu L, Zhang X, Kao R, Kalbfleisch J, Williams D, Li C (2016). MicroRNA-214 protects against hypoxia/reoxygenation induced cell damage and myocardial ischemia/reperfusion injury via suppression of PTEN and Bim1 expression. Oncotarget.

[ref-65] Wang L, Kang FB, Shan BE (2014). B7-H3-mediated tumor immunology: friend or foe?. International Journal of Cancer.

[ref-66] Wang P, Li ZW, Zhu Z, Zhang ZY, Liu J (2019a). Inhibition of miR-214-5p attenuates inflammatory chemotaxis and nerve regeneration obstruction after spinal cord injury in rats. European Review for Medical and Pharmacological Sciences.

[ref-67] Wang CJ, Zhu CC, Xu J, Wang M, Zhao WY, Liu Q, Zhao G, Zhang ZZ (2019b). The lncRNA UCA1 promotes proliferation, migration, immune escape and inhibits apoptosis in gastric cancer by sponging anti-tumor miRNAs. Molecular Cancer.

[ref-68] Watanabe T, Sato T, Amano T, Kawamura Y, Kawamura N, Kawaguchi H, Yamashita N, Kurihara H, Nakaoka T (2008). Dnm3os, a non-coding RNA, is required for normal growth and skeletal development in mice. Developmental Dynamics.

[ref-69] Wu D, Lu P, Mi X, Miao J (2018a). Exosomal miR-214 from endometrial stromal cells inhibits endometriosis fibrosis. Molecular Human Reproduction.

[ref-70] Wu K, Ma J, Zhan Y, Liu K, Ye Z, Chen J, Xu K, Huang H, He Y (2018b). Down-regulation of microrna-214 contributed to the enhanced mitochondrial transcription factor A and inhibited proliferation of colorectal cancer cells. Cellular Physiology and Biochemistry.

[ref-71] Xiao Y, Guo L, Zhao S, Huang G, Chen S, Yang L, Li Y, Li B (2019). MiR-214 regulates CD3*ζ* expression in T cells. Central European Journal of Immunology.

[ref-72] Xie Y, Ma X, Chen L, Li H, Gu L, Gao Y, Zhang Y, Li X, Fan Y, Chen J, Zhang X (2017). MicroRNAs with prognostic significance in bladder cancer: a systematic review and meta-analysis. Scientific Reports.

[ref-73] Yan Z, Zang B, Gong X, Ren J, Wang R (2020). MiR-214-3p exacerbates kidney damages and inflammation induced by hyperlipidemic pancreatitis complicated with acute renal injury. Life Sciences.

[ref-74] Yanagawa-Matsuda A, Kitamura T, Higashino F, Yamano S, Totsuka Y, Shindoh M (2012). E1A expression might be controlled by miR-214 in cells with low adenovirus productivity. Virus Research.

[ref-75] Yang H, Kong W, He L, Zhao JJ, O’Donnell JD, Wang J, Wenham RM, Coppola D, Kruk PA, Nicosia SV, Cheng JQ (2008). MicroRNA expression profiling in human ovarian cancer: miR-214 induces cell survival and cisplatin resistance by targeting PTEN. Cancer Research.

[ref-76] Yin Y, Cai X, Chen X, Liang H, Zhang Y, Li J, Wang Z, Chen X, Zhang W, Yokoyama S, Wang C, Li L, Li L, Hou D, Dong L, Xu T, Hiroi T, Yang F, Ji H, Zhang J, Zen K, Zhang CY (2014). Tumor-secreted miR-214 induces regulatory T cells: a major link between immune evasion and tumor growth. Cell Research.

[ref-77] Zhang C, Li N, Fu X, Lin Q, Lin L, Tu J (2019). MiR-214 inhibits snakehead vesiculovirus (SHVV) replication by targeting host GS. Fish and Shellfish Immunology.

[ref-78] Zhang Y, Liu D, Chen X, Li J, Li L, Bian Z, Sun F, Lu J, Yin Y, Cai X, Sun Q, Wang K, Ba Y, Wang Q, Wang D, Yang J, Liu P, Xu T, Yan Q, Zhang J, Zen K, Zhang CY (2010). Secreted monocytic miR-150 enhances targeted endothelial cell migration. Molecular Cell Cell.

[ref-79] Zhang J, Medeiros LJ, Young KH (2018). Cancer immunotherapy in diffuse large B-cell lymphoma. Frontiers in Oncology.

[ref-80] Zhang KC, Xi HQ, Cui JX, Shen WS, Li JY, Wei B, Chen L (2015). Hemolysis-free plasma miR-214 as novel biomarker of gastric cancer and is correlated with distant metastasis. American Journal of Cancer Research.

[ref-81] Zhang C, Yi L, Feng S, Liu X, Su J, Lin L, Tu J (2017). MicroRNA miR-214 inhibits snakehead vesiculovirus replication by targeting the coding regions of viral N and P. Journal of General Virology.

[ref-82] Zhao L, Liu YW, Yang T, Gan L, Yang N, Dai SS, He F (2015). The mutual regulation between miR-214 and A2AR signaling plays an important role in inflammatory response. Cellular Signalling.

[ref-83] Zhao Y, Lin Q, Li N, Babu VS, Fu X, Liu L, Liang H, Liu X, Lin L (2019). MicroRNAs profiles of Chinese Perch Brain (CPB) cells infected with Siniperca chuatsi rhabdovirus (SCRV). Fish and Shellfish Immunology.

[ref-84] Zheng D, Zang Y, Xu H, Wang Y, Cao X, Wang T, Pan M, Shi J, Li X (2019). MicroRNA-214 promotes the calcification of human aortic valve interstitial cells through the acceleration of inflammatory reactions with activated MyD88/NF-*κ*B signaling. Clinical Research in Cardiology.

[ref-85] Zhou Y, Tian W, Zhang M, Ren T, Sun G, Jiang R, Han R, Kang X, Yan F (2019). Transcriptom analysis revealed regulation of dexamethasone induced microRNAs in chicken thymus. Journal of Cellular Biochemistry.

